# Genome Sequence of *Streptomyces aureofaciens* ATCC Strain 10762

**DOI:** 10.1128/genomeA.00615-16

**Published:** 2016-06-23

**Authors:** Julien S. Gradnigo, Greg A. Somerville, Michael J. Huether, Richard J. Kemmy, Craig M. Johnson, Michael G. Oliver, Etsuko N. Moriyama

**Affiliations:** a School of Biological Sciences, University of Nebraska–Lincoln, Lincoln, Nebraska, USA; b School of Veterinary Medicine and Biomedical Sciences, University of Nebraska–Lincoln, Lincoln, Nebraska, USA; c Zoetis, Lincoln, Nebraska, USA; d Center for Plant Science Innovation, University of Nebraska–Lincoln, Lincoln, Nebraska, USA

## Abstract

*Streptomyces aureofaciens* is a Gram-positive actinomycete that produces the antibiotics tetracycline and chlortetracycline. Here, we report the assembly and initial annotation of the draft genome sequence of *S. aureofaciens* ATCC strain 10762.

## GENOME ANNOUNCEMENT

*Streptomyces aureofaciens* was first identified in 1948 (1), from Plot 23 in Sanborn Field, a timothy hayfield at the University of Missouri (2). Although it has been used for the commercial production of tetracycline antibiotics (1, 3) and has been the subject of numerous biochemical studies, a genome assembly of *S. aureofaciens* was not publicly available. Here, we report the draft assembly of the whole-genome sequence of the *S. aureofaciens* ATCC strain 10762 and its initial annotation.

*S. aureofaciens* ATCC strain 10762 was cultivated in a chemically defined medium [4] and grown for 9 days at 30°C with 150-rpm aeration. High-molecular-weight DNA was prepared from fresh cultures by cetyltrimethylammonium bromide extraction (5). Genomic DNA was evaluated for molecular weight integrity by agarose gel electrophoresis and nucleic acid fluorometric quantitation for construction of the DNA library.

Genome sequencing and read quality filtering were done by Eurofins MWG Operon (Alabama, USA). Illumina MiSeq sequencing was done with long-jumping-distance sequencing (3-kb and 8-kb inserts), generating paired-end 150-bp reads. After removing very short (<30 bp) reads, adapter-trimming, and quality-clipping using Trimmomatic (6), 2.46 Gb of sequence information in 19.42 million reads (3.90 million pairs and 12.84 million singletons) were obtained. Shotgun sequencing on the Roche 454 Genome Sequencer FLX platform produced 132.76 Mb of sequence data in 209,530 reads with a mean length of 633 bp after trimming with Trimmomatic.

Genome assembly was done using SPAdes version 3.7.1 with the “--careful” option (to reduce mismatches and short indels) (7) with Illumina (both paired-end and singleton) and 454 reads, resulting in 120 contigs (total length: 9,234,994 bp; maximum length: 881,164 bp; *N*_50_: 228,235 bp) in 60 scaffolds (total length: 9,244,380 bp; maximum length: 1,746,076 bp; *N*_50_: 660,648 bp). The average G+C content was 71.2%.

Genome annotation was done using the NCBI Prokaryotic Genome Annotation Pipeline version 3.1 (8). In total, 13 contigs were removed from the annotation because they were <200 bp or were of non–*S. aureofaciens* origin (e.g., plasmid). From the remaining 107 contigs, a total of 8,083 genes were annotated. This includes 7,541 protein-coding genes, 22 rRNA genes (5S, 16S, and 23S), 72 tRNA genes, and 445 potential pseudogenes.

Four related *S. aureofaciens* strains were recently deposited in the NCBI Assembly database (ASM71917v1, ASM97851v1, ASM71688v1, and ASM127066v1; these strains were designated NRRL B-2657, NRRL 2209, NRRL B-1286, and NRRL B-2658, respectively). Our assembled contigs are virtually identical to ASM71917v1 (99.75% total aligned bases using MUMmer version 3.23 [9]) and only slightly divergent from ASM97851v1 (99.05% total aligned bases). Our assembly has a greater total length and *N*_50_ and a smaller number of contigs compared to these assemblies. Interestingly, our assembled contigs differ significantly from ASM71688v1 and ASM127066v1 (83.69% and 9.92% total aligned bases, respectively).

### Nucleotide sequence accession numbers.

This whole-genome shotgun project of *S. aureofaciens* ATCC strain 10762 has been deposited in DDBJ/ENA/GenBank under the accession number JPRF00000000. The version described in this paper is the second version, JPRF02000000.
